# First Case of FLT3-Tyrosine Kinase Domain Mutant Acute Myeloid Leukemia With Unusual Onset as Isolated Bilateral Testicular Myeloid Sarcoma

**DOI:** 10.7759/cureus.58140

**Published:** 2024-04-12

**Authors:** Veysel Erol, Gulsum Akgun Cagliyan, Furkan Ufuk, Derya Demir

**Affiliations:** 1 Depatment of Hematology, Kahramanmaras Necip Fazil City Hospital, Kahramanmaras, TUR; 2 Department of Hematology, Pamukkale University Hospital, Denizli, TUR; 3 Department of Radiology, Pamukkale University Hospital, Denizli, TUR; 4 Department of Pathology, Ege University Faculty of Medicine, Izmir, TUR

**Keywords:** flt-3 mutation, midostaurine, systemic chemotherapy, testicular myeloid sarcoma, acute myeloid leukemia

## Abstract

Testicular myeloid sarcoma (TMS) is a challenging pathology often posing diagnostic difficulties due to the poorly differentiated nature of tumor cells at the initial presentation. The delay in diagnosis significantly impacts patient life expectancy, emphasizing the need for prompt identification and treatment initiation. In certain cases, the presence of the Fms-like tyrosine kinase (*FLT3*) mutation adds complexity to the disease, requiring tailored therapeutic approaches. In this report, we present a unique case of bilateral TMS with *FLT3* tyrosine kinase domain (*TKD*) mutation. The patient exhibited an aggressive clinical course, initially misdiagnosed with orchitis during the initial evaluation. Subsequent reevaluation of the testicular biopsy at a second center led to an accurate diagnosis, highlighting the importance of thorough examination in challenging cases. Given the emerging significance of *FLT3* mutations in myeloid sarcomas, comprehensive testing for all *FLT3* variants is crucial to determine the appropriate treatment modality. This case underscores the need for increased awareness among healthcare professionals regarding the diagnostic nuances and potential genetic variations associated with TMS. Furthermore, the inclusion of tyrosine kinase inhibitors, such as midostaurin or gilteritinib, especially in the presence of *FLT3* mutations, may significantly impact treatment outcomes.

This report contributes to the growing body of literature on TMS and highlights the importance of considering *FLT3* mutations in the diagnostic and therapeutic decision-making process for improved patient care.

## Introduction

Myeloid sarcoma is a type of solid tumor that involves immature myeloid precursors originating from myeloid hematopoietic cells and often involves extra-bone marrow tissues [1]. Myeloid sarcoma may occur in association with acute myeloid leukemia (AML) or blastic transformations of myeloproliferative diseases, such as chronic myeloid leukemia, polycythemia vera, and myelofibrosis [2,3]. Myeloid sarcoma can be present as a clinical manifestation in approximately 1-8% of AML cases [4,5]. While isolated myeloid sarcoma may occur with bone, periosteum, soft tissue, and lymph node involvement, involvements of the orbit, intestinal region, mediastinum, epidural region, and ovaries are less common [6]. Myeloid sarcoma can develop simultaneously with AML or during relapse, long before the onset of AML symptoms, or less frequently as isolated myeloid sarcoma cases. The majority of isolated myeloid sarcoma cases consist of acute myelomonocytic leukemia, acute monocytic leukemia, and chronic myelomonocytic leukemia [7].

FMS-like tyrosine kinase-3 (*FLT3*) is a type 3 receptor tyrosine kinase belonging to the tyrosine kinase group, and it is detected in approximately 90% of AML cases. *FLT3* mutation is present in about 30% of AML cases [8]. The internal tandem duplication (*ITD*) mutation is the most common variant of *FLT3*, accounting for approximately 30% of all AML cases [9,10]. A high *FLT3-ITD* mutant allele burden to normal *FLT3-ITD* ratio is associated with poor prognosis. The *FLT3*-tyrosine kinase domain (*TKD*) mutation is the second most common mutation variant and is found in approximately 14% of all AML cases [11]. The impact of *FLT3-TKD* mutation allele burden rates on the prognosis for AML is still subject to debate [12].

Testicular myeloid sarcoma (TMS) is a rare form of myeloid sarcoma, and most cases are seen in the form of isolated unilateral testicular involvement. However, TMS cases are often associated with involvement in other areas, such as lymph nodes, skin, orbit, and soft tissue. As of 2021, only four out of 68 reported cases of TMS had bilateral isolated testicular involvement [13]. In this case report, we present the treatment and follow-up of the first case in the literature, which is an *FLT3-TKD* mutant AML patient who had a history of isolated bilateral testicular involvement as the initial manifestation.

## Case presentation

A 51-year-old male patient visited the urology clinic four months ago due to bilateral scrotal swelling. The patient was treated with oral antibiotics with a preliminary diagnosis of orchitis but was readmitted one month later as the swelling did not improve. Heterogeneity in the bilateral testicles with abnormally increased vascularity was shown on Doppler ultrasonographic evaluation. According to ultrasonographic evaluation, radical orchiectomy was planned by a urologist, but the procedure was denied by the patient. After this, a testicular biopsy was performed in November 2022, and the diagnosis was inconclusive between T-cell lymphoma, lymphoblastic lymphoma, and myeloid sarcoma. Since a definitive diagnosis could not be made, the testicular biopsy sample was sent to another center for a second. The patient was discharged with a recommendation of control with biopsy results after clinical, hemogram, and biochemical tests were not life-threatening. The patient’s hemogram and serum chemistry values before discharge were normal, except LDH, which was 651 U/L. One month later, the patient presented to the emergency department with widespread body pain, weakness, an increase in scrotal pain, and confusion.

**Figure 1:**
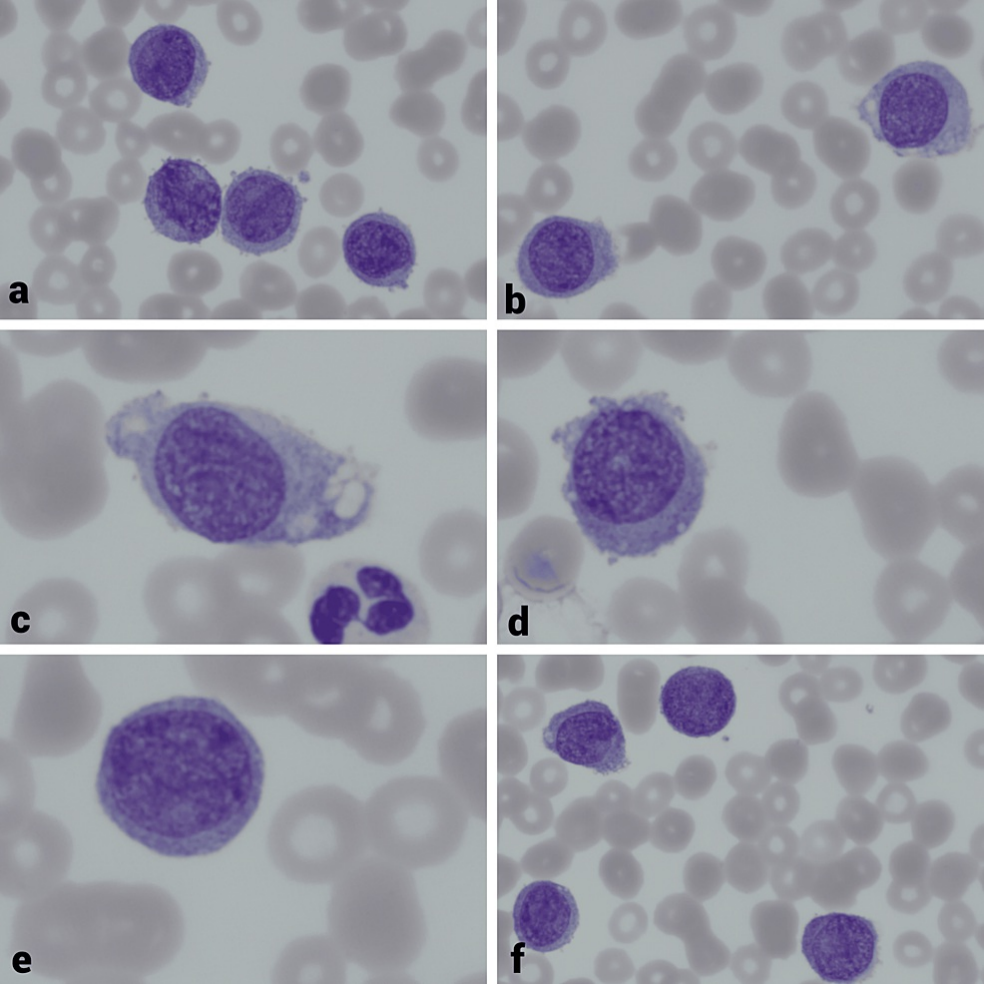
(a)-(f) Blasts with rounded nuclei, loose chromatin network, and basophilic cytoplasm in the M5A variant according to FAB.

The patient was referred to the hematology clinic with the pathology results. The patient’s laboratory results on admission are given in Table 1. It was found to have widespread monoblastic blasts in the peripheral smear evaluation (Figure 1).

**Table 1:** Laboratory parameters of the patient on admission. AST, aspartate aminotransferase; ALT, alanine aminotransferase; LDH, lactate dehydrogenase; CRP, C-reactive protein

Parameters	Patient results (on admission)	Normal range (units)
White blood count	50,000 K/µL	4000-10,000 K/µL
Neutrophil	21,000 K/µL	2000-7000 K/µL
Hemoglobin	12.3 g/dL	12-18 g/dL
Thrombocyte	53,000 K/µL	100,000-38,0000 K/µL
Creatine	1.07 mg/L	0.7-1.2 mg/L
AST	29 IU/L	<40 IU/L
ALT	14 IU/L	<41 IU/L
LDH	2743 U/L	135-225 U/L
CRP	207 mg/L	<5 mg/L

**Figure 2:**
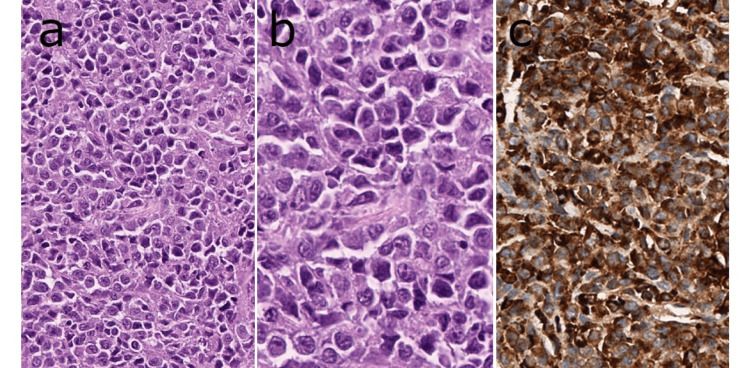
The tumor was characterized by diffuse infiltration of blastic cells (a) with prominent nucleoli and (b) a high mitotic index. (c) Tumor cells were diffusely positive for CD68-KP1 immunohistochemically.

A diffuse blastic cell infiltration was detected in the histopathological examination of the testicular biopsy in the second evaluation. Immunohistochemically, CD4, CD33, CD43, CD45, CD56, CD68-KP1, and Bcl-2 were common and strong positive (Figure 2); CD38 and CD68-PGM1 were pale positive; CD163 was focal positive; and CD2, CD3, CD5, CD7, CD8, OCT2, PAX5, CD79a, CD138, CD23, Bcl-6, CD10, CD34, CD117, CD123, CD30, CD99, MPO, Lysozyme, OCT4, TdT, TCL1, TCR-beta, EMA, cyclin D1, MUM1, HHV8, synaptophysin, chromogranin-A, desmin, and pancytokeratin negativity were detected. CD15 could not be assessed due to suboptimal samples, and the Ki-67 proliferation index was found to be around 85%. EBV-encoded RNA (EBER) was negative in chromogenic in situ hybridization examination. According to these findings, a TMS diagnosis was made.

**Figure 3:**
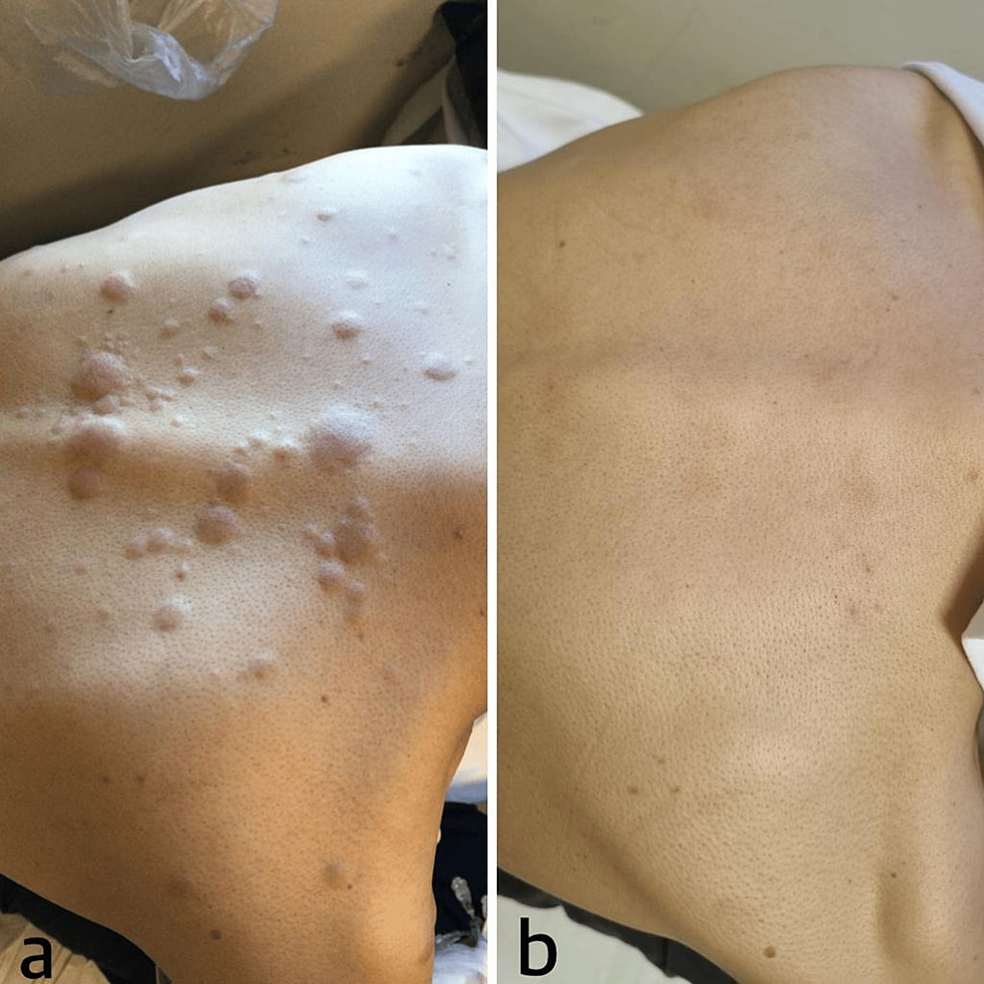
(a) Maculopapular rash lesions extending to the neck bilaterally at the level of the scapula before the treatment. (b) Skin findings resolved after chemotherapy.

**Figure 4:**
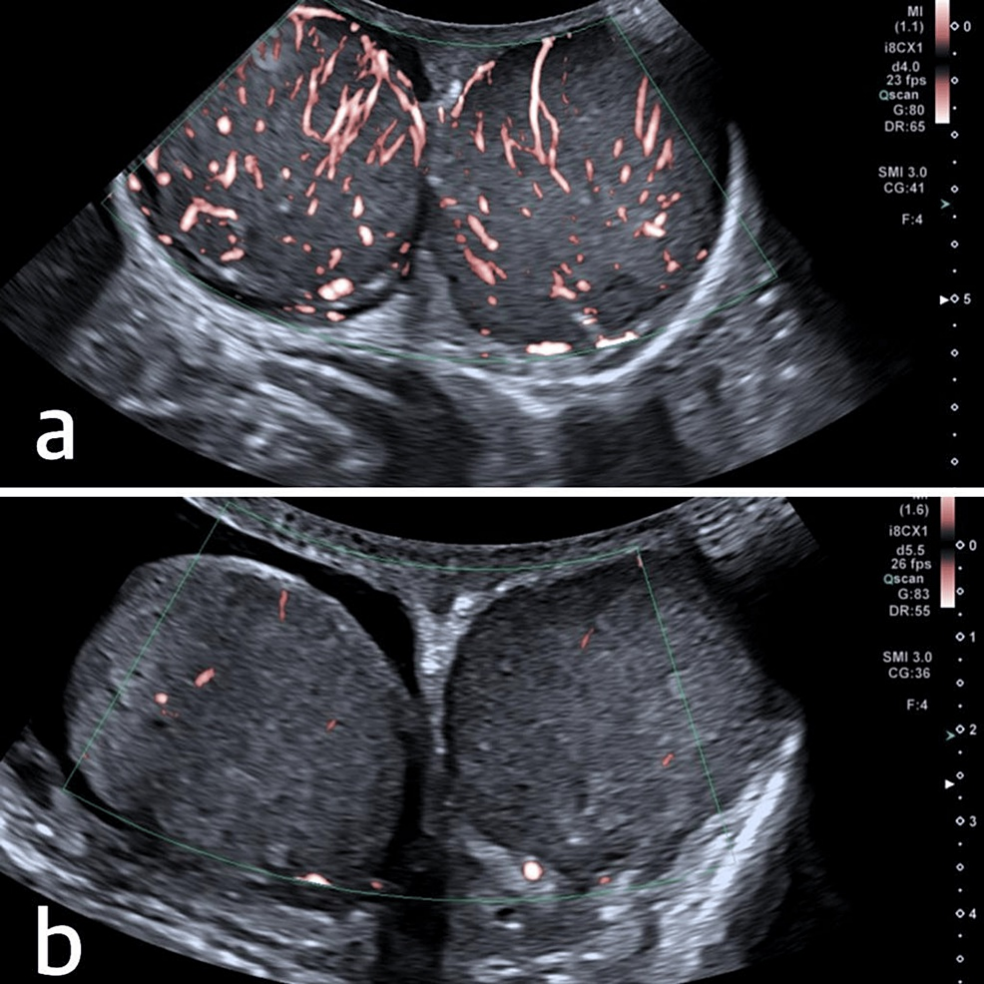
(a) Scrotal DUS image showing heterogeneity in the bilateral testicles with abnormally increased vascularity. (b) Post-treatment scrotal DUS image demonstrating the regression of vascularity in the bilateral testicles. DUS, Doppler ultrasound

A bone marrow biopsy-aspiration was performed, and extensive blastic infiltration that suppressed the entire hematopoietic series was detected in the bone marrow aspiration evaluation. The patient was diagnosed with AML-NOS, monoblastic leukemia (M5B), in line with WHO classification. CD13, CD33, and CD64 were widely positive, while CD34 and MPO were weakly positive in flow cytometry evaluation. The patient underwent a diagnostic lumbar puncture due to the high-risk nature of the disease, and cytology was reported as normal. A brain magnetic resonance imaging (MRI) was performed to investigate the patient’s confusion, but no abnormalities were detected. The patient had diffuse, non-pruritic maculopapular lesions on the bilateral scapula and neck region, and a skin biopsy was performed with a preliminary diagnosis of leukemic involvement, yet only mononuclear infiltration was observed. Since the tumor cells of the testes were highly undifferentiated and there was no other secondary pathology to explain the skin lesions, we considered the patient’s skin findings as leukemic involvement (Figure 3). In addition, a scrotal Doppler ultrasound (DUS) was performed due to scrotal swelling, which showed heterogeneity in the bilateral testicles with increased vascularity (Figure 4a). Furthermore, neck and body computed tomography (CT) scans were obtained, revealing conglomerating lymphadenopathies in the neck, retroperitoneum, and inguinal region, as well as splenomegaly.

The patient received cytarabine 230 mg plus idarubicin 23 mg chemotherapy and 12.5 mg intrathecal methotrexate treatment on a weekly basis for a total of four weeks following diagnostic procedures. Bone marrow biopsy-aspiration performed on the 33rd day of chemotherapy confirmed remission. Control scrotal DUS was performed, and vascularity regression in the bilateral testicles was shown (Figure 4b). Three weeks after chemotherapy, leukemia-related molecular genetic studies, nucleophosmin (*NPM1*), *FLT3*, and CCAAT-enhancer-binding protein alpha (*CEBPA*) showed *FLT3-TKD* mutations in 68% of cells. As a result, midostaurin 2 × 50 mg was added to the patient’s high-dose cytarabine 2 × 5000 mg consolidation treatment. AML-related cytogenetic studies were normal. The *FLT3-TKD* allele level was reduced to 0 after the first course of consolidation chemotherapy, and the patient’s general condition and laboratory results were good, with the maintenance of remission status. Currently, the patient is receiving the second course of consolidation treatment, and donor screening for an allogeneic stem cell transplant is in progress. This case highlights a rare occurrence of *FLT3-TKD* mutant isolated bilateral TMS after admission with AML.

## Discussion

A myeloid sarcoma is a rare form of AML whose incidence and total number of cases are not well established due to the lack of data in the literature. Histopathologically, myeloid sarcoma can be confused with diseases such as high-grade lymphoma, plasmablastic lymphoma, melanoma, Ewing’s sarcoma, and blastic plasmacytoid dendritic cell neoplasia [3]. Approximately half of the cases are reported as misdiagnoses at the time of diagnosis [5]. A study by Goyal et al. has identified 746 myeloid sarcoma cases according to the 10-year National Cancer Network Database search, of which only 43 cases were reported as related to the reproductive system [14]. Although reproductive myeloid sarcoma can occur in any age range, most cases occur 10 years prior to the myeloid sarcoma of other systems. The prognosis of TMS cases, relative to other system myeloid sarcoma cases, is still debated, with some studies indicating no difference in prognosis, while others suggest a better prognosis due to earlier findings [15,16]. However, it has been reported that isolated TMS cases had a worse prognosis compared to other AML cases [17]. Our case was a newly diagnosed *FLT3-TKD* mutant with bilateral testicular involvement, suggesting that *FLT3-TKD* mutation can be observed in TMS cases at the time of diagnosis.

Although there is no clear protocol for the treatment of TMS cases, treatment typically involves surgery, radiotherapy (RT), systemic chemotherapy, and hypomethylating agent therapy. Since the testicles are immune-privileged areas and the blood-testicular barrier has selective permeability to chemotherapy agents, the risk of recurrence after treatment is high. Due to the aggressive recurrence following focal treatments, such as RT, systemic chemotherapy, similar to AML type regimen, is currently the most effective treatment for isolated TMS cases [18]. In cases of *FLT3-TKD* mutant TMS, *FLT3* tyrosine kinase inhibitors (midostaurin and quizartinib) can be added to the treatment, as they have a positive effect on overall survival. After achieving remission, the referral of the patient for allogeneic bone marrow transplantation, in line with their age, performance, and comorbidities, is the main focus of the treatment. *FLT3* allele load can be followed to predict early recurrence in these cases.

## Conclusions

TMS cases can present with symptoms that may be confused with infective processes or other neoplasms, making histopathological evaluation of the testicular biopsy specimen crucial for accurate diagnosis and effective treatment. Given the aggressive nature of these cases, treatment should be initiated promptly upon diagnosis. *FLT3-ITD* mutation has been detected in cases to date, and the presence of *FLT3-TKD* mutation must not be overlooked, as in our case. Although systemic chemotherapy and allogeneic stem cell transplantation are currently the most effective treatment approaches, the addition of midostaurin to the treatment plan can significantly enhance the treatment response of these aggressive cases in the presence of *FLT3* mutation.
